# Chitosan from Mushroom Improves Drought Stress Tolerance in Tomatoes

**DOI:** 10.3390/plants13071038

**Published:** 2024-04-06

**Authors:** Olusoji Demehin, Maha Attjioui, Oscar Goñi, Shane O’Connell

**Affiliations:** 1 Plant Biostimulant Group, Shannon Applied Biotechnology Centre, Munster Technological University-Tralee (South Campus), Clash, V92CX88 Tralee, Co. Kerry, Ireland; o.demehin@marigot.ie (O.D.); maha.att@gmail.com (M.A.); shane.oconnell@mtu.ie (S.O.); 2 Marigot Ltd., Marigot Research Center, Sycamore Court, V92N6C8 Tralee, Co. Kerry, Ireland; 3 Brandon Bioscience, Marigot Research Center, Sycamore Court, V92N6C8 Tralee, Co. Kerry, Ireland

**Keywords:** chitin, biopolymers, biostimulant, crustacean, abiotic stress, proline, malondialdehyde, soluble sugars, plants, yield

## Abstract

Chitosan is a derivative of chitin that is one of the most abundant biopolymers in nature, found in crustacean shells as well as in fungi cell walls. Most of the commercially available chitosans are produced from the exoskeletons of crustaceans. The extraction process involves harsh chemicals, has limited potential due to the seasonal and limited supply and could cause allergic reactions. However, chitosan has been shown to alleviate the negative effect of environmental stressors in plants, but there is sparse evidence of how chitosan source affects this bioactivity. The aim of this study was to investigate the ability of chitosan from mushroom in comparison to crustacean chitosan in enhancing drought stress tolerance in tomato plants (cv. MicroTom). Chitosan treatment was applied through foliar application and plants were exposed to two 14-day drought stress periods at vegetative and fruit set growth stages. Phenotypic (e.g., fruit number and weight), physiological (RWC) and biochemical-stress-related markers (osmolytes, photosynthetic pigments and malondialdehyde) were analyzed at different time points during the crop growth cycle. Our hypothesis was that this drought stress model will negatively impact tomato plants while the foliar application of chitosan extracted from either crustacean or mushroom will alleviate this effect. Our findings indicate that drought stress markedly decreased the leaf relative water content (RWC) and chlorophyll content, increased lipid peroxidation, and significantly reduced the average fruit number. Chitosan application, regardless of the source, improved these parameters and enhanced plant tolerance to drought stress. It provides a comparative study of the biostimulant activity of chitosan from diverse sources and suggests that chitosan sourced from fungi could serve as a more sustainable and environmentally friendly alternative to the current chitosan from crustaceans.

## 1. Introduction

The agricultural sector is faced with threats that are negatively impacting crop production. These threats are caused by abiotic stressors resulting from climate change and human activities. Abiotic stresses in plants are mediated by several factors such as drought, elevated temperatures, salinity, toxic metal ions, and radiation caused by ultraviolet rays [1]. However, drought is an abiotic stress that requires urgent attention as the world is facing water supply limitations in highly productive agricultural regions [2]. Drought refers to an imbalance between rainfall and evapotranspiration in a particular region over a prolonged period [3]. A prolonged period of absence of rainfall and/or lack of irrigation leads to water deficit in plants, which negatively impacts plant health. The seriousness of this impact is usually uncertain owing to its dependency on the occurrence and rainfall distribution, moisture storing capacity and evaporative demands in soils [4]. Most losses recorded in agricultural production occur during the vegetative stage because of random drought conditions and sometimes during the reproductive stage of plants because of extreme drought stress events [5].

The impact of drought is evident on several plant phenotypic, biochemical, and molecular parameters. Drought stress usually induces stomata closure, which in turn affects CO_2_ uptake by leaves and causes a reduction in photosynthetic enzymatic activity, leading to reduced photosynthesis [6]. Drought stress also causes a reduction in the water potential, relative water content (RWC) and plant transpiration, usually leading to increased leaf temperature [7]. Moreover, the availability, uptake, translocation, and metabolism of nutrients from root to aboveground organs are affected by water deficit [6]. Therefore, the result of drought stress at the phenotype level is characterized by impairment of seed germination [8] and a reduction in plant growth and yield [6]. Plants can activate different biochemical and molecular responses against drought stress. These responses include the biosynthesis of osmolytes such as proline [1], accumulation of phytohormones such as abscisic acid [9] and jasmonic acid [10], or activation of scavenging mechanisms of reactive oxygen species (ROS) [11]. The scavenging response to the presence of ROS can be enzymatic or non-enzymatic. The enzymatic response includes the production of enzymes such as superoxide dismutase, monodehydroascorbate reductase, catalase, dehydroascorbate reductase and glutathione reductase, guaiacol peroxidase, ascorbate peroxidase, etc. The non-enzymatic response includes the synthesis of proline, ascorbic acid (AA), flavonoids, carotenoids, glutathione (GSH), and α-tocopherol [11]. In combating and alleviating the threat posed to agriculture through drought stress, methods such as irrigation, plant breeding, genetic modification, and the use of drought-resistant crops are mostly employed [12]. In this regard, a promising strategy is the use of substances known as biostimulants. Biostimulants are substance(s) and/or microorganisms whose function when applied to plants or the rhizosphere is to mediate natural processes that will enhance/benefit nutrient use efficiency, tolerance to abiotic stress, and crop quality traits [13]. Biostimulants can be classified based on their source material and mode of action. These categories include humic substances, microbial inoculants, seaweed extracts, chitin and chitosan derivatives, protein hydrolysates and other N-containing substances [14].

Chitosan is a derivative of chitin generated through a process known as deacetylation [15]. Chitin is naturally found in the exoskeleton of crustaceans, arachnids, insects, and fungi but not in higher organisms and vertebrates [16]. Due to its non-toxicity, availability, solubility and reported bioactivities, there have been continuous efforts to explore multiple applications of chitosan in various fields, especially agriculture as a plant biostimulant. Studies have shown that chitosan can enhance tolerance against drought stress in plants by scavenging ROS, enhancing water absorption capacity by increasing the root length, and heightening photosynthetic activities [17]. Another study on the reduction in transpiration by chitosan in pepper (*Capsicum* spp.) [18] recorded a 26–43% reduction in water usage by plants treated with chitosan without altering biomass production and yield. Chitosan also has been shown to increase the proline level and reduce lipid peroxidation in drought-stressed thyme, leading to the preservation of cell membrane integrity [19]. Similarly, a significant rise in lipid peroxidation was recorded by [12] in drought-stressed tomato plants. They also recorded a downregulation in the level of proline and sucrose in control drought stress tomato plants compared to those treated with a commercial biostimulant. In another study [20], it was demonstrated that drought stress reduces the height, number of leaves, chlorophyll content and RWC of tomato plants. However, the tomato plants showed more tolerance to drought stress when treated with chitosan. Chitosan and its derivatives were able to induce tolerance in maize under drought stress by increasing the activity of antioxidant enzymes that reduce the oxidative stress caused by ROS accumulation [21].

Currently, most of the commercially available chitosan used in studies that focus on mitigation of drought stress are produced from crustacean chitin. However, crustaceans are subjected to seasonal availability and could be a source of allergens and heavy metals such as nickel and copper; strong chemical treatment is also required for its extraction [22]. Therefore, it is preferable to find an alternative source of chitin that can be used later to generate chitosan. Mushrooms, for example, can be a safer, sustainable, and non-animal source of chitin and chitosan [23]. Our hypothesis was that drought stress will negatively impact overall plant productivity while the foliar application of different chitosan sources will alleviate this effect. Tomato was chosen as a model plant system for this study as it is one of the widely cultivated crops worldwide and is useful whether as a fresh or processed product. Specifically, we worked with the cultivar MicroTom as it is a widely applied laboratory model among tomato cultivars because of its shorter growth cycle and smaller size [24]. The comparative study of the biostimulant activity of chitosan from diverse sources suggests that biostimulant formulations based on mushroom chitosan could serve as a sustainable and environmentally friendly alternative to alleviate the negative effects of drought stress on tomato productivity.

## 2. Results

### 2.1. Effect of Chitosan on Final Fruit Yield and Weight in Tomato Plants under Drought Stress

The two 14-day drought stress periods resulted in significant differences between unstressed and stressed plants (Table 1; Figure S1). However, foliar application of chitosan was able to alleviate the impact of drought on the fruit number. Additionally, drought stress reduced the average fruit weight (Table 1), while no statistically significant impact of chitosan on the average fruit weight was recorded. The fruit number, pre-anthesis, active flowers and post-anthesis in the plants prior to stress and chitosan application (T0) are presented in Table S1 in the supplementary section, confirming the presence of a homogenous phenotype in the different groups before starting the experiment.

### 2.2. Effect of Chitosan on RWC, Proline, and Soluble Sugar Content of Tomato Leaves under Drought Stress

As shown in Table 2, drought stress significantly reduced RWC in all treatments during the first stress period (T1). In the stressed group, the application of crustacean chitosan (CC) significantly increased the RWC compared to the stressed control and stressed plants treated with mushroom chitosan (MC). Drought stress also significantly increased proline accumulation in stressed groups compared to unstressed groups (Table 2). Foliar application of MC during the first drought stress period significantly increased the proline level in the drought-stressed group compared to the stressed Ctrl and the stressed CC. However, no difference was observed between stressed Ctrl and stressed CC. In the unstressed group, the proline content of unstressed MC was significantly higher than unstressed Ctrl, but no statistically significant difference was observed between unstressed Ctrl and unstressed CC. In addition, the sucrose level was higher in the stressed group compared to the unstressed group, and significant differences were also observed between treatments, while MC was higher than Ctrl, and no difference was observed between the two chitosan groups and between Ctrl and CC. 

At the end of the first recovery stage (T2), there were no significant differences in RWC between groups and treatments. However, there was a significant difference between the two groups in terms of proline accumulation and sucrose. Moreover, statistically significant differences were observed within the stressed group; both chitosan treatments showed a lower accumulation of proline compared to the Ctrl, but no difference was found between unstressed treatments. At the end of the second drought stress period (T3), a significant difference between stressed and unstressed plants was observed in terms of RWC, proline and sucrose content. In the stressed plants, MC-treated plants showed a higher RWC compared to stressed Ctrl and stressed CC. In terms of proline, there was a difference between treatments and MC had a higher proline level compared to the Ctrl and CC, but the Ctrl and CC treatments were similar. After the last recovery stage (T4), RWC in stressed plants was significantly lower than unstressed plants. Proline and sucrose levels were significantly high in drought-stressed plants compared to unstressed plants. Plants treated with chitosan showed similar RWC compared to untreated plants under drought stress. Plants treated with MC showed significantly elevated levels of proline under drought stress conditions compared to the Ctrl, while no differences were observed between Ctrl and CC and between MC and CC. In terms of sucrose, no difference was observed between unstressed Ctrl and unstressed MC, but unstressed CC was higher than both unstressed Ctrl and MC, while in the stressed group, MC was higher than Ctrl and CC. The RWC, proline and soluble sugar content prior to stress and chitosan treatment (T0) are presented in the supplementary section (Table S2), confirming the absence of statistically significant differences between groups before starting the experiment.

### 2.3. Effect of Chitosan on MDA, Total Chlorophyll, and Carotenoids Content in Tomato Leaves under Drought Stress

The level of oxidative stress in the plants was measured in terms of the malondialdehyde (MDA) content and impact on photosynthetic pigments (Table 3). Drought stress significantly increased the level of MDA in stressed plants compared to those unstressed at T1 and T3. During these two stress periods (T1 and T3), drought stress also led to the significant accumulation of total chlorophyll in plants compared to those unstressed with drought (Table 3). The carotenoids content was not significantly affected by drought application (Table 3). In the first drought stress period (T1), foliar application of chitosan to plants under stress had no considerable influence on MDA and chlorophyll and carotenoids content. However, in the second drought period (T3), the application of mushroom chitosan significantly reduced the MDA level, while both chitosan significantly increased the chlorophyll content. During the first recovery stage (T2), drought stress significantly increased the MDA level, while it significantly reduced the chlorophyll content. However, plants treated with both chitosan under drought stress showed a reduced but not significant MDA level, while those treated with crustacean chitosan showed a significantly high chlorophyll content. Plants treated with mushroom chitosan also showed a reduced carotenoids compared to those treated with crustacean’s chitosan and control.

In the second recovery stage (T4), drought stress had no significant impact on MDA, chlorophyll, and carotenoids content in plants. However, plants treated with chitosan had an MDA content that was significantly higher than that of control plants under drought stress. The levels of malondialdehyde, chlorophyll and carotenoids prior to stress and chitosan treatment (T0) are presented in the supplementary section (Table S2), confirming, as above, the absence of statistically significant differences between groups before starting the experiment.

## 3. Discussion

Plants are usually exposed to various stresses during their life cycle, which affects their metabolic activities, growth, and development. Amid these stresses, drought stress tends to be the most severe to plant productivity. Plant fresh biomass is 80–95% water, which is important for numerous physiological and metabolic processes needed for plant growth and development [25]. In an era of increasing water scarcity [2] due to climate change, finding a sustainable and environmentally friendly approach to alleviate the effect of drought stress in plants is important. The use of plant biostimulants, a group of substances applied to plants to trigger processes that enhance plant tolerance to abiotic stressors, seems to be a promising approach. Chitosan, a derivative of chitin, is a biostimulant that has been shown to enhance plant tolerance against some stress conditions such as drought [17,18,19,21]. However, most of the chitosan whose defense activities have been reported in literature are from a marine source. Therefore, in this study, we investigated the ability of chitosan from a mushroom source to enhance plant tolerance against drought stress in comparison to chitosan of marine origin. Analysis was performed to understand the mode of action of chitosan on a phenotypic, biochemical, and physiological level in plants. In general, drought stress usually impairs plant productivity, as evident in the final fruit number and yield. In this study, drought stress reduced the average final fruit number by 19% between stressed and unstressed plants. However, foliar application of mushroom and crustacean chitosan to the drought-stressed plant group increased the average fruit number by 32% and 17%, respectively (Table 1). While drought stress also reduced the final fruit weight by 35%; foliar application of crustacean and mushroom chitosan to the drought-stressed plant group increased it by 1% and 3%, respectively. These results suggest that foliar application of mushroom chitosan enhances plant productivity like marine-sourced chitosan under drought conditions, although these changes are not statistically significant at *p* ≤ 0.05. In a recent study [20], it was shown that drought stress significantly reduced the final tomato fruit number and weight. Additionally, their results showed that foliar application of chitosan at 0.001% *w*/*v* significantly increased the fruit number and weight with respect to the control. Overall, our results indicate that mushrooms could be a potential source for chitosan generation and enhance tomato plants’ tolerance to the drought stress condition, further improving the fruit number and weight of treated drought-stressed plants.

### 3.1. Effect of Chitosan on RWC in Tomato Plants under Drought Stress

RWC is the principal indicator of the water level in plants as it mirrors the parity between water supply to leaf tissue at a period of sampling and the actual amount the leaf can hold [26]. During the two stages of drought stress (T1 and T3), stressed plants showed a significant decrease in RWC compared to the unstressed plant group (Table 2). This is like the findings of Wu et al., 2017 [27], Farooq et al., 2009 [6], Soltys-Kalina et al., 2016 [26], and Hassnain et al., 2020 [20]. However, foliar application of chitosan to drought-stressed plants increased their RWC as compared to stress control. In the first drought stage (T1), the RWC of plants under drought stress and treated with crustacean chitosan was significantly increased while a similar significant increment was recorded in plants treated with mushroom chitosan during the second drought stress (T3). Chitosan was also reported to significantly increase RWC in drought-stressed tomato plants [20] and in maize [28]. Some studies attributed this to the ability of chitosan to expand the cell layer and improve antioxidant activities, which further influenced the water holding capacity in plants under stress conditions. There was no observable impact of chitosan application on the plant RWC at the end of each recovery stage (T2 and T4). These results reflect the effectiveness of chitosan, regardless of its source, in enhancing the leaf water retention of tomato plants during drought stress.

### 3.2. Effect of Chitosan on Proline Content in Tomato Plants under Drought Stress

The accumulation of proline in plants is associated with a lower water potential of plant tissues, thus preventing water loss, and enhancing water uptake from the soil environment [19]. While Hong et al., 2000 [29], described proline accumulation as a well-known response of drought-stressed plants, Hayat et al., 2012 [1] indicated that the accumulation of proline is not a suitable marker to measure the level of drought stress as other abiotic stresses happening simultaneously could also trigger its production. This could be a result of the usual correlation with levels of RWC both in treated and untreated plants under drought stress. As observed in this study, the proline level in leaf tissue was significantly higher in drought-stressed tomato plants compared to unstressed plants. Plants under the drought stress condition showed a 4-fold and 15-fold increase in proline level compared to unstressed plants at T1 and T3, respectively (Table 2). This elevated level of proline in stressed plants was also recorded in thyme plants under drought by Emami et al., 2017 [19], in safflower by Mahdavi et al., 2011 [30], and in tomato plants by Goñi et al., 2018 [12]. However, foliar application of chitosan to plants under drought stress enhanced further the synthesis of this osmoregulator. The level of proline in plants treated with crustacean chitosan was significantly higher during the first stage of stress (T1), while a non-significant increment was recorded in those treated with mushroom chitosan. However, during the second drought stage (T3), plants treated with mushroom chitosan have a higher level of proline compared to those treated with crustacean chitosan. Foliar application of chitosan to drought-stressed plants has been reported to induce the accumulation of proline in maize [21], safflower [30] and thyme [19]. Proline is an important osmoprotectant responsible for reducing ROS levels; thus, its accumulation during stress is crucial to plant health. This shows that the level of proline is usually higher in stress-tolerant than stress-sensitive plants [12].

### 3.3. Effect of Chitosan on Soluble Sugar Content in Tomato Plants under Drought Stress

The level of soluble sugars such as sucrose is also reported as a marker for drought stress tolerance. Soluble sugars, like proline, are linked to antioxidant defense, stress signaling, osmotic adjustment, and energy metabolism during stress [31]. Many studies have associated their accumulation with stress tolerance in plants. In the present study, the accumulation of sucrose during each phase was significantly high in drought-stressed plants compared to unstressed plants. This indicates the physiological struggle of the plants to generate energy and solutes for osmoprotection. A report by Rosa et al., 2009 [32], suggested that sucrose either functions as a substrate for cellular respiration or as an osmolyte to sustain cell turgor during stress. The level of sucrose triggered by mushroom chitosan was significant in the first stage of drought stress compared to control plants. However, both chitosan sources triggered higher production of sucrose during the second drought stage (T3), but this was not significant compared to the control under stress. Transcriptome evidence has revealed that some genes responsible for carbohydrate metabolism, energy production, ascorbate-glutathione and flavonoid metabolism are usually upregulated by chitosan during drought stress [31]. Other studies have also shown that foliar application of chitosan induces the upregulation of sucrose in maize [21] and a non-significant accumulation in thyme [19]. However, there was no evidence that chitosan application impacted sucrose accumulation at the end of each recovery stage. The ability of mushroom chitosan to induce the upregulation of sucrose in this study is further evidence of its defense-eliciting ability like marine-sourced chitosan.

### 3.4. Effect of Chitosan on Photosynthetic Pigment in Tomato Plants under Drought Stress

The effect of drought stress on plants’ photosynthetic pigments can be heterogenous. Some studies reported that drought stress decreases [33,34,35] or increases [36] photosynthetic pigment such as chlorophyll. This disparity could be because of the plant type and experimental procedure used for analysis [37]. During the two-drought stress periods (T1 and T3), drought-stressed plants showed a significant reduction in chlorophyll content compared to unstressed plants. This reduction was also significant at the end of the first recovery stage. Which could be an indication of plants’ sensitivity to stress at the early stage of their growth cycle. This was not the case at the end of the second recovery stage when plants were older. The level of carotenoids was not significantly affected by drought stress. The reduction in the chlorophyll level recorded in the stressed plants in this study is similar to the findings of Hassnain et al., 2020 [20]; they reported a significant decrease in chlorophyll content of tomato plants under twelve days of severe drought. Foliar application of mushroom and crustacean chitosan increased the chlorophyll content, though this was not significant among the plant group in the first drought stage while a statistically significant effect was recorded in the second stage of stress. The increment of photosynthetic pigment in plants under stress due to foliar application of chitosan as observed in this study agrees with the findings of Hassnain et al., 2020 [20], and Emami et al., 2017 [19]. At the end of the last recovery stage, a non-statistically significant increase in the chlorophyll and decrease in carotenoids content were recorded in plants treated with chitosan under drought stress. These analyses indicate that chitosan from mushroom and marine sources have a similar influence on the photosynthetic pattern of tomato plants under drought stress.

### 3.5. Effect of Chitosan on Malondialdehyde (MDA) in Tomato Plants under Drought Stress

Under stress, plants stimulate the production of reactive oxygen species (ROS), which impairs the production of essential biomolecules such as proteins, nucleic acids, and lipids [37]. The level of membrane lipid damage caused by ROS in plants is measured by the amount of malondialdehyde (MDA) in a plant tissue. In this study, drought stress significantly upregulated the level of MDA in stressed plants compared to the unstressed plant group. These results are consistent with reports from the literature [18,38]. The foliar application of crustacean and mushroom chitosan has no impact on the MDA level in the stressed plant group during the first drought stage. However, a reduction in the MDA level was recorded for plants treated with chitosan in the stressed group during the second stage of drought application. There are scarce data on the effect of chitosan on the MDA content of tomato plants under drought stress. However, a study on *Thymus daenensis* [19] reported that the application of chitosan to drought-stressed thymes caused a reduction in MDA content. Similar reports on *Carthamus tinctorius* were given by Madhavi et al., 2011 [30], indicating that chitosan lowered the MDA content of safflower treated with a low concentration of chitosan. The ability of chitosan to reduce the antioxidant activity of reactive oxygen species has been associated with chitosan-abundant active hydroxyl and amino groups. These functional groups on chitosan can form stable and non-toxic macromolecular complexes with ROS [39]. The abundance of these functional groups in both chitosan formulations because of their similar degree of deacetylation could explain the similarity in their biostimulant activities as recorded in this study.

### 3.6. Conclusions

A clear impact of drought stress on the phenotypic, biochemical, and physiological traits of plants was observed in this study. Plants subjected to the two 14-day water deficit stresses showed a significant decrease in the average number and weight of final fruits. A reduction in the level of RWC coupled with a significant increase in lipid peroxidation measured in terms of the MDA content in drought-stressed plants provided a basis for this effect. However, foliar application of crustacean and mushroom chitosan improved the physiological water status and increased the level of osmolytes, consequently improving the fruit yield and quality. These are consistent with findings from the literature on the impact of drought and chitosan treatment on plant productivity. Nevertheless, plants treated with mushroom chitosan performed better in inducing a higher accumulation in tomato of osmoprotectants such as proline and sucrose compared to the effect observed with crustacean-chitosan-treated plants. These findings also showed that chitosan from mushroom possesses a similar ability to crustacean chitosan in increasing an important yield marker in tomato plants (fruit number). This experimental evidence suggests that biostimulant formulations based on mushroom chitosan can have a role in improving fruit set under drought stress conditions. However, to expand the applicability and impact of mushroom chitosan biostimulants in the agricultural sector, further investigation supporting its efficacy in other important horticulture crops at the field level will be important.

## 4. Materials and Methods

### 4.1. Materials

Mushroom chitosan (MC) DDA = 60% was generated in our laboratory through the deacetylation of chitin obtained from white button mushroom (*Agaricus bisporus*). Its degree of deacetylation was analyzed using potentiometric titration [40] and the average viscosity molecular weight (Mv) was determined using the Mark–Houwink equation [15]. A crustacean chitosan (CC) was used as a positive control and was kindly donated by the research group of Prof. Dr. Bruno M. Moerschbacher from the University of Münster, Germany. Tomato seeds (cv. MicroTom) were purchased from Moles Seeds (Essex, UK).

### 4.2. Experimental Design

**Figure 1:**
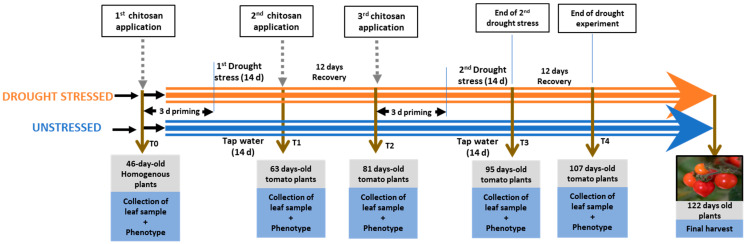
Workflow diagram of drought stress treatment, chitosan application and sampling. Priming involves the treatment of plants with chitosan or control solution prior to start of drought stress.

Crustacean chitosan (CC) and mushroom chitosan (MC) of similar DDA were dissolved in a weak acetic acid solution. Tomato seeds (cv. MicroTom) were grown in a soil composed of compost/vermiculite/perlite (6:1:1) in standard plug trays for four weeks before transplanting into a similar medium in 2 L pots. The plants were grown in a growth room at a temperature of 27/22 ± 1°C with 16 h of daylight, 8 h of night and 80 ± 5% RH under a light intensity of 120 μmol m^−2^ s^−1^. Plants were grouped in a 2 × 3 completely randomized block design into stressed and unstressed groups. Stressed and unstressed groups were further divided into control, mushroom chitosan (MC) and crustacean chitosan (CC) sub-groups with 8 plants per sub/group. After transplanting (T0), 0.01% *w*/*v* chitosan (MC and CC) was applied to 46-day-old plants through foliar spray to prime the plants for 3 days prior to the start of drought stress (Figure 1). The control group was sprayed with solvent (acetic acid in water). Three days after the first foliar application of chitosan, 49-day-old plants were subjected to drought stress by not watering them for 14 days (T1, 63-day-old plants). At the end of the first drought stress period (T1), plants were re-watered, and a 2nd chitosan treatment was applied 3 days later. Afterwards, the plants were left in the recovery stage for an additional 12 days (T2). A 3rd chitosan treatment was applied to 78-day-old plants at the end of the first recovery stage to prime the plants for a second drought stress. After 72 h, plants were subjected to a second drought stress for 14 days (T3, 95-day-old plants). At the end of the second drought stress, plants were re-watered and left in recovery for 12 days (T4, 107 days old plants). Fruits were harvested from 122-day-old plants for weight and quality analysis. Plant tissues were sampled at different time points before the foliar application (T0), at the end of the first drought stress (T1), end of first recovery stage (T2), at the end of the second drought stress (T3), and at the end of second recovery stage (T4). At each sampling point, leaf tissues (3 leaves per plant, including old and young leaf) were taken and snap-frozen in liquid nitrogen, kept in −80 °C until further analysis. The level of significance of the impact of drought and chitosan treatment on fruit number and fruit weight were evaluated by comparing means of fruits obtained from eight plants per treatment subgroup (stress control, MC, and CC or unstressed control, MC, and CC). Measured biochemical and physiological parameters such as RWC, proline, MDA, chlorophyll, carotenoids, and sucrose are data from three biological replicates (randomly sampled from the subgroup) and three technical replicates per biological replicates.

### 4.3. Phenotypic Evaluation of Tomato Plants and Fruit Yield Assessment

Prior to the first application of chitosan and drought stress, the flowering pattern of the plants was evaluated. Additionally, at the end of the trial, the fruit number and yield were measured considering both ripened and unripe fruits.

### 4.4. RWC in Leaf Tissue

Leaf samples were taken before treatments and four sampling points during the trial and were analyzed for their RWC as follows. The fresh weight (FW) of each leaf was measured immediately after cutting. To obtain the turgid weight (TW), the leaf samples were soaked in distilled water at room temperature in darkness for 3 h. After imbibition, the excess of water in the leaves was carefully removed and leaves were weighed. To measure the dry weight (DW), the leaf samples were transferred to an oven and dried at 80 °C overnight before weighing. The percentage RWC was calculated as follows [12]:

%RWC = [(FW − DW)/(TW − DW)] × 100

### 4.5. Chlorophyll and Carotenoids Content in Leaf Tissue

As previously described by Goñi et al., 2018, and Lichenthaler et al., 2001 [12,41], leaf samples taken during each sampling period were snap frozen and grinded into powder. A measure of 100 mg of the ground leaf samples was extracted in 80% acetone at 4 °C for 2 h, centrifuged at 20,000× *g* for 10 min at 4 °C. The resulting supernatants were collected and diluted with 80% acetone before absorbance reading. Absorbance at 664 nm and 647 nm was measured. The following equations [12,41] were used for the determination of total chlorophyll (as sum of chlorophyll a + chlorophyll b) and content of carotenoids:

Chlorophyll a (Ca) = 12.25 × A664 − 2.79 × A647

Chlorophyll b (Cb) = 21.50 × A647 − 5.10 × A664

Carotenoids = [1000 × A470 − (1.82 × Ca − 85.02 × Cb)]/198

Results for the content of photosynthetic pigments are expressed as µg/DW.

### 4.6. Proline Content in Leaf Tissue

The proline content was measured according to the method described by Goñi et al., 2018 [12]. Briefly, 0.5 mL of 70% ethanol was added to 50 mg of leaf sample and left in the dark overnight at 4 °C. The supernatant was obtained afterwards by centrifuging at 20,000× *g* for 10 min at 4 °C. A total of 400 µL of a mixture of 1% *w*/*v* ninhydrin in acetic/ethanol/water (60/20/20) was added to 200 µL of the supernatant and incubated at 95 °C for 20 min. Absorbance was read at 520 nm. Results were expressed as mg/g(DW) equivalent of L-proline standards.

### 4.7. Malondialdehyde Content (MDA) in Leaf Tissue

The MDA content in leaf samples was measured according to the method described by Hodegs et al., 1999 [42]. To measure leaf samples’ MDA content, 0.5 mL of 80% ethanol was added to 50 mg of sample and incubated at 4 °C for 1 h. Supernatants were obtained after centrifugation at 20,000× *g* for 10 min at 4 °C and divided into two equal aliquots. Each aliquot was mixed with 1) a mixture of 20% (*w*/*v*) trichloroacetic acid (TCA) and 0.5% (*w*/*v*) thiobarbituric acid (TBA) or 2) a pure solution of 20% (*w*/*v*) trichloroacetic acid. The tubes were mixed and incubated at 95 °C for 40 min. Afterwards, samples were cooled and centrifuged at 2000× *g* for 5 min at 4 °C. The absorbance of the supernatant was read at 440 nm, 532 nm and 600 nm. The MDA content was calculated using equations reported by [42]. Results are expressed as nmol/mgDW.

### 4.8. Sucrose Content

Sucrose in leaf tissue was analyzed as described by Goñi et al., 2018 [12]. The extraction of soluble sugars from leaf tissues was performed by adding 2% (*w*/*v*) polyvinylpyrrolidone (PVPP) to 15 mg of leaf sample and incubating at 90 °C for 25 min. The extract was sonicated before centrifugation to obtain the supernatant of soluble sugars at 15,000 rpm at 4 °C for 20 min. The soluble sugars were separated on a Carbopac PA-1 column in the presence of a 90 mM NaOH as mobile phase at a 1 mL/min flow rate. Sucrose detection was performed using a pulsed amperometric detector (PAD) and compared to the analytical grade standard (Sigma-Aldrich, Arklow, Ireland). Results are expressed as mg/gDW.

### 4.9. Statistical Analysis

Except otherwise stated, all data are the average and standard deviation of replicates. The levels of significance of the effect of drought stress (D) and foliar application of chitosan (C) on all plant parameters were evaluated by comparing means of data with two-way ANOVA and Tukey’s HSD test (*p* ≤ 0.05) using Sigma Plot v.12 software. Where the interaction (D × C) between the two-factor condition (D) and treatment (C) was significant, data were subjected to one-way ANOVA and Tukey’s HSD test at *p* ≤ 0.05. The effect of drought and chitosan treatment was evaluated separately as well, comparing the respective means through either a *t*-test at *p* ≤ 0.05 or one-way ANOVA and Tukey’s HSD test at *p* ≤ 0.05. The application of all parametric tests was performed after checking the data normality (Shapiro–Wilk test) and equal variance assumptions (ANOVA *p* ≤ 0.05).

## Figures and Tables

**Table 1:** Effect of chitosan on the final fruit number and weight of tomato plants under drought stress.

Treatments	Number of Fruits	Fruits Weight (g)
Drought (D)		
Unstressed	15.38 b	35.58 b
Stressed	12.38 a	22.99 a
Chitosan (C)		
Control	11.679 a	29.57
MC	14.223 ab	26.5
CC	15.732 b	31.78
D × C		
Unstressed × Ctrl	12.88	36.50
Unstressed × MC	14.57	29.69
Unstressed × CC	18.71	40.55
Stressed × Ctrl	10.50	22.64
Stressed × MC	13.88	23.31
Stressed × CC	12.75	23.01
Statistical significance		
Drought (D)	*	**
Chitosan (C)	*	ns
D × C	ns	ns

Results are average of sample replicates at the end of trial. ns, *, **, means non-significant, statistically significant differences at *p* ≤ 0.05, *p* ≤ 0.01 and *p* ≤ 0.001, respectively. Different letters within each parameter indicate statistically significant differences within the same factor (Drought, Chitosan or Drought × Chitosan). The analysis is based on two-way ANOVA analysis and Tukey’s HSD post hoc test. Ctrl: control, MC: mushroom chitosan, CC: crustacean chitosan. Fruit numbers and weight (*n* ≥ 8).

**Table 2:** Effect of chitosan on RWC, proline, soluble sugar content of tomato leaves under drought stress.

Treatments	T1			T2			T3			T4		
	%RWC	Proline (mg/gDW)	Sucrose (mg/gDW)	%RWC	Proline (mg/gDW)	Sucrose (mg/gDW)	%RWC	Proline (mg/gDW)	Sucrose (mg/gDW)	%RWC	Proline (mg/gDW)	Sucrose (mg/gDW)
Drought (D)												
Unstressed	91.79 b	2.08 a	20.38 a	88.9	0.95 a	19.24 a	89.65 a	1.28 a	17.64 a	82.89 b	0.34 a	16.36 a
Stressed	78.60 a	10.72 b	51.14 b	90.06	4.4 b	24.85 b	61.45 b	20.53 b	41.08 b	77.55 a	4.99 b	23.37 b
Chitosan (C)												
Control	83.02 a	5.56 a	33.31 a	88.69	3.3 b	22.22	71.83 a	10.24 a	28.3	80.11	2.41	19.04
MC	84.26 a	8.06 b	37.53 b	90.56	2.27 a	20.60	79.69 b	11.64 b	30.34	79.76	2.79	20.71
CC	88.31 b	5.58 a	36.45 ab	89.2	2.45 a	23.33	75.13 a	10.84 a	29.44	80.79	2.81	19.85
D × C												
Unstressed × Ctrl	90.99	1.62 a	18.89	88.99	0.7 a	18.51	89.62 c	1.22	18.72	83.00	0.40 a	15.97 a
Unstressed × MC	91.26	2.4 b	20.52	89.83	0.7 a	18.66	89.46 c	1.39	16.92	82.02	0.12 a	12.94 a
Unstressed × CC	93.12	2.22 ab	21.73	87.89	1.35 a	20.55	89.86 c	1.22	17.2	83.65	0.51 a	20.18 b
Stressed × Ctrl	75.05 a	9.5 c	47.73	88.39	5.90 c	25.92	54.05 a	19.26	37.89	77.22	4.42 b	22.10 b
Stressed × MC	77.27 a	13.72 d	54.53	91.29	3.76 b	22.53	69.91 b	21.89	43.76	77.49	5.46 c	28.47 c
Stressed × CC	83.50 b	8.93 c	51.17	90.51	3.55 b	26.12	60.40 a	20.46	41.60	77.93	5.11 bc	19.52 b
Statistical significance												
Drought (D)	***	***	***	ns	***	***	***	***	***	***	***	***
Chitosan (C)	***	***	*	ns	***	ns	***	*	ns	ns	ns	ns
D × C	*	***	ns	ns	***	ns	***	ns	ns	ns	**	***

Results are means of sample replicates at each time point. ns, *, **, *** means non-significant, statistically significant differences at *p* ≤ 0.05, *p* ≤ 0.01 and *p* ≤ 0.001, respectively. Different letters within each parameter indicate statistically significant differences within the same factor (Drought, Chitosan or Drought × Chitosan). The analysis is based on two-way ANOVA analysis and Tukey’s HSD post hoc test. Ctrl; control, MC; mushroom chitosan, CC; crustacean chitosan. Number of biological replicates (*n* ≥ 3).

**Table 3:** Effect of chitosan on MDA, total chlorophyll, and carotenoids content in tomato leaves under drought stress.

Treatments	T1			T2			T3			T4		
	MDA (nmol/mgDW)	Chlorophyll (µg/DW)	Carotenoids (µg/DW)	MDA (nmol/mgDW)	Chlorophyll (µg/DW)	Carotenoids (µg/DW)	MDA (nmol/mgDW)	Chlorophyll (µg/DW)	Carotenoids(µg/DW)	MDA (nmol/mgDW)	Chlorophyll (µg/DW)	Carotenoids (µg/DW)
Drought (D)												
Unstressed	3.37 a	30.38 b	2.93	1.46 a	35.86 b	3.611	3.03 a	28.84 b	3.05	3.78	26.41	2.66
Stressed	4.39 b	28.1 a	3.06	2.09 b	32.11 a	3.47	3.62 b	26.57 a	3.11	4.05	26.89	2.66
Chitosan (C)												
Control	3.79	27.86	2.8	1.72	34.03 ab	3.49	3.75 b	25.61 a	3.01	3.45	26.64	2.67
MC	3.96	30.24	3.12	1.8	32.13 a	3.43	3.32 ab	28.43 b	3.15	4.04	26.12	2.57
CC	3.89	29.62	2.99	1.8	35.79 b	3.70	2.91 a	29.07 b	3.08	4.25	27.19	2.74
D × C												
Unstressed × Ctrl	3.45 ab	27.72	2.64	1.3	34.72 b	3.38 ab	3.29	28.46 b	2.97	3.89 ab	26.36	2.59
Unstressed × MC	3.7 ab	31.73	3.06	1.53	37.2 b	3.80 b	2.91	29.2 b	3.15	3.61 ab	25.95	2.64
Unstressed × CC	2.95 a	31.68	3.09	1.55	35.60 b	3.65 b	2.89	28.79 a	3.03	3.83 ab	26.91	2.74
Stressed × Ctrl	4.14 bc	27.99	3.10	2.14	33.3 b	3.6 b	4.1	22.75 b	3.04	3.01 a	26.91	2.76
Stressed × MC	4.21 bc	28.75	3.18	2.07	27.0 a	3.0 a	3.7	27.6 b	3.14	4.4 ab	26.3	2.49
Stressed × CC	4.82 c	27.56	2.91	2.05	35.99 b	3.7 b	2.93	29.34 b	3.13	4.67 b	27.47	2.73
Statistical significance												
Drought (D)	***	*	Ns	***	***	ns	**	*	ns	ns	ns	ns
Chitosan (C)	Ns	Ns	Ns	ns	*	ns	**	*	ns	ns	ns	ns
D × C	*	Ns	Ns	ns	***	***	ns	*	ns	*	ns	ns

Results are means of sample replicates at each time point. ns, *, **, *** means non-significant, statistically significant differences at *p* ≤ 0.05, *p* ≤ 0.01 and *p* ≤ 0.001, respectively. Different letters within each parameter indicate statistically significant differences within the same factor (Drought, Chitosan or Drought × Chitosan). The analysis is based on two-way ANOVA analysis and Tukey’s HSD post hoc test. Ctrl; control, MC; mushroom chitosan, CC; crustacean chitosan. Number of biological replicates (n ≥ 3).

## Data Availability

Data is contained within the article and Supplementary Materials.

## Supplementary Materials

The following are available online at https://www.mdpi.com/article/10.3390/plants13071038/s1, Figure S1: Pictures of marketable fruits at the end of drought stress trial; Table S1: Phenotypic and physiologic parameters and RWC before application of chitosan and drought stress (T0); Table S2: Biochemical parameters before application of chitosan and drought stress (T0).
