# Supplementary material for: Chitosan from Mushroom Improves Drought Stress Tolerance in Tomatoes
Source: Plants (Basel). 2024 Apr 6;13(7):1038. doi: 10.3390/plants13071038 (PMC11013739; doi:10.3390/plants13071038)
Supplement: Supplementary file 1 [file plants-13-01038-s001.zip › plants-2857421-supplementary.pdf]

Supplementary Materials:

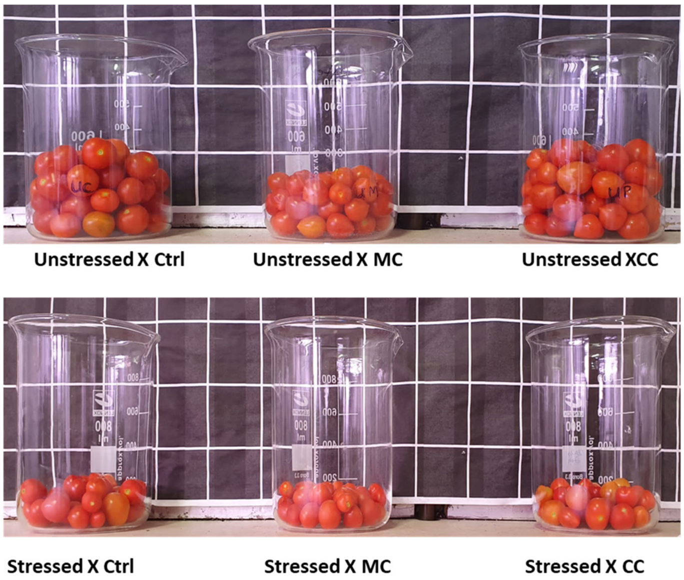

Figure S1. Marketable fruits at the end of drought stress trial.

Table S1 Phenotypic and physiologic parameters and relative water content before application of chitosan and drought stress (T0)

Treatments	Pre-anthesis	Active flowers	Post anthesis	Fruiting	%RWC
Unstressed x Ctrl	7.5±2.9 a	0.5	0	0	80.22±3.5 a
Unstressed x MC	5.5±2.5 a	0	0	0	84.04±3.9 a
Unstressed x CC	5.5±1.2 a	0	0	0	83.64±4.9 a
Stressed x Ctrl	9.75±1.19 a	0	0	0	84.33±2.0 a
Stressed x MC	9.13±1.5 a	0	0	0	84.79±1.8 a
Stressed x CC	8.63±1.9 a	0	0	0	89.55±0.7 a

Different letters within each parameter indicate statistically significant differences within the same factor (Stressed or unstressed). The analysis is based on one-way ANOVA analysis turkey HSD post hoc test. Ctrl; Control, MC; Mushroom chitosan, CC; Commercial chitosan.

Table S2. Biochemical parameters before application of chitosan and drought stress.

Treatments	Chlorophyll (µg/DW)	Carotenoids (µg/DW)	Proline (%DW)	Sucrose (mg/gDW)	MDA (nmol/mgDW)
Unstressed x Ctrl	33.48±4.9 a	1.37±0.7 a	4.04±1.3 a	18.58±1.8 b	22.17±3.4 b
Unstressed x MC	44.76±12.3 a	1.54±1.0 a	3.77±1.4 a	10.45±1.0 a	16.34±3.2 a

Unstressed x CC	44.76±12.3 a	1.54±1.0 a	3.77±1.4 a	10.45±1.0 a	16.34±3.2 a
Stressed x Ctrl	33.35±3.0 a	1.74±0.1 a	3.93±0.5 b	17.55±1.1 a	19.07±3.3 a
Stressed x MC	32.66±3.9 a	1.67±0.4 a	2.13±0.4 a	18.74±1.5 a	22.17±3.3 a
Stressed x CC	32.66±3.9 a	1.67±0.4 a	2.13±0.4 a	18.74±1.5 a	22.17±3.3 a

Different letters within each parameter indicate statistically significant differences within the same factor (Stressed or unstressed). The analysis is based on one-way ANOVA analysis turkey HSD post hoc test. Ctrl; Control, MC; Mushroom chitosan, CC; Commercial chitosan.
